# New Hygien of the Mouth

**Published:** 1854-07

**Authors:** 


☞ We received, a short time since, the following card from a professional friend, and give it to the profession as the *latest improvement.* We are now more than convinced that we "are not familiar with *all* the wonderful mysteries of the new Hygien of the mouth."—Ed.

"Dr. James Taylor—*Dear Sir* :—I enclose a *professional card*, which, aside from its literary merits, will convince you, I think, that you are not familiar with *all* the wonderful mysteries of the "new Hygien of the Mouth,"—the art of "embalming of teeth" for instance! But let us reverently stand aside when the brilliant Professor from "Paree" advances! Look! listen! *learn!*

"As for myself, I can at least say that I have seen the "specimens,"—the "said 'Osanore,' without any springs nor hooks," whose ghastly pallor confronted the street-passer. Rude carvings of dead-white *ivory*, the bases highly stained with some red decoction, constituted that "perfect similarity." But as for the other peculiarities of this "new Hygien," I cannot attempt any elucidation.

"Yours, &c., C. T. C."

---

### New Hygien of the Mouth.

EMBALMING AND MASTICATION OF TEETH.

By the aid of that new System, the extraction which is a dreaded operation becomes useless. The most violent toothache is instantly stopped

### BY THE DOCTOR GARRAUD,

*Dentist of the Hospitals of Paris.*

Mr. Garraud, sets the teeth said "Osanore," without any springs nor hooks. In one word, Mr. Garraud practices the art of Dentistry in all its branches. The above said is preferable in every respect for mastication, and for the most perfect similarity, so much so that Dentists themselves could not find any difference between the natural and artificial teeth. The mouth can be opened without any fear, and the natural motions of the mouth can be made without showing any ligatures or hooks. When those hooks keep moving in the mouth, it is painful; beside, they corrode the teeth upon which they rest.

Mr. Garraud would respectfully invite the Ladies and Gentlemen who wish to honor him with their confidence to call at his residence and examine his specimens. *All the above said and his work are guaranteed.*

---

### INCOMPARABLE ANTISPASMODIC ELIXIR.

Those who would follow the advice of the author of that preparation may be sure not to have to use the services of any Dentist. That preparation prevent the diseases of the mouth.

To prevent any counterfeit of the afore said preparation, the signature of the author will be on every bottle.

**DR. GARRAUD, 412 Broadway.**

DEAR EDITOR :—I am treating myself to a ramble among the profession, of which I may tell you some day. Some items are decidedly rich.

The following is from my friend Dr. Messenger, who had changed his location, after having prepared a mouth for the insertion of the four incisors. He informed his patron by letter of his new location, which was as convenient for him as the old one. He received in reply the following, *verbatim, et literatim, et punctuatim.* The names only are suppressed :

Sep the 6 . . 1853

Sir i recevd your letter the third Statting that you would like for me to cum to mt vernon to get you to due the Job i had bin to n . . . . . to Se you an had thot i would cum to get you to due the Job but i have met with a nether man to due the Job which Ses he will do it for Six dollars an in Shore it so i thot that was cheeper than you would due it

So remaines yours ——— ———

